# Ranolazine-Mediated Attenuation of Mechanoelectric Feedback in Atrial Myocyte Monolayers

**DOI:** 10.3389/fphys.2020.00922

**Published:** 2020-08-04

**Authors:** Irene Del-Canto, Lidia Gómez-Cid, Ismael Hernández-Romero, María S. Guillem, María Eugenia Fernández-Santos, Felipe Atienza, Luis Such, Francisco Fernández-Avilés, Francisco J. Chorro, Andreu M. Climent

**Affiliations:** ^1^INCLIVA Health Research Institute, Centro de Investigación Biomédica en Red en Enfermedades Cardiovasculares, Valencia, Spain; ^2^Department of Electronic Engineering, Universitat Politècnica de València, Valencia, Spain; ^3^Department of Cardiology, Hospital General Universitario Gregorio Marañón, Instituto de Investigación Sanitaria Gregorio Marañón, Centro de Investigación Biomédica en Red en Enfermedades Cardiovasculares, Madrid, Spain; ^4^Department of Signal Theory and Communications, Universidad Rey Juan Carlos, Madrid, Spain; ^5^ITACA Institute, Universitat Politècnica de València, Valencia, Spain; ^6^Department of Physiology, Universitat de València Estudi General, Valencia, Spain; ^7^Department of Cardiology, Hospital Clínico Universitario de Valencia, INCLIVA, Valencia, Spain

**Keywords:** mechanical stretch, mechanoelectric feedback, fibrillatory patterns, ranolazine, optical mapping, rotor dynamic analysis, HL-1 cell

## Abstract

**Background:**

Mechanical stretch increases Na^+^ inflow into myocytes, related to mechanisms including stretch-activated channels or Na^+^/H^+^ exchanger activation, involving Ca^2+^ increase that leads to changes in electrophysiological properties favoring arrhythmia induction. Ranolazine is an antianginal drug with confirmed beneficial effects against cardiac arrhythmias associated with the augmentation of *I*_NaL_ current and Ca^2+^ overload.

**Objective:**

This study investigates the effects of mechanical stretch on activation patterns in atrial cell monolayers and its pharmacological response to ranolazine.

**Methods:**

Confluent HL-1 cells were cultured in silicone membrane plates and were stretched to 110% of original length. The characteristics of *in vitro* fibrillation (dominant frequency, regularity index, density of phase singularities, rotor meandering, and rotor curvature) were analyzed using optical mapping in order to study the mechanoelectric response to stretch under control conditions and ranolazine action.

**Results:**

HL-1 cell stretch increased fibrillatory dominant frequency (3.65 ± 0.69 vs. 4.35 ± 0.74 Hz, *p* < 0.01) and activation complexity (1.97 ± 0.45 vs. 2.66 ± 0.58 PS/cm^2^, *p* < 0.01) under control conditions. These effects were related to stretch-induced changes affecting the reentrant patterns, comprising a decrease in rotor meandering (0.72 ± 0.12 vs. 0.62 ± 0.12 cm/s, *p* < 0.001) and an increase in wavefront curvature (4.90 ± 0.42 vs. 5.68 ± 0.40 rad/cm, *p* < 0.001). Ranolazine reduced stretch-induced effects, attenuating the activation rate increment (12.8% vs. 19.7%, *p* < 0.01) and maintaining activation complexity—both parameters being lower during stretch than under control conditions. Moreover, under baseline conditions, ranolazine slowed and regularized the activation patterns (3.04 ± 0.61 vs. 3.65 ± 0.69 Hz, *p* < 0.01).

**Conclusion:**

Ranolazine attenuates the modifications of activation patterns induced by mechanical stretch in atrial myocyte monolayers.

## Introduction

Mechanical stretch is an arrhythmogenic factor in different cardiovascular disorders such as arterial hypertension, mitral valve disease, and congestive heart failure, as well as in acute clinical scenarios such as pulmonary embolism, acute heart failure, acute valve regurgitation, hypertensive crises, or the initial moments of tachyarrhythmia (Ravelli and Allessie, 1997; De Jong et al., 2011; Jalife, 2011; Strege et al., 2012). Although the electrophysiological effects of mechanical stretch on atrial myocytes have been described in previous works (Ravelli et al., 2011; Strege et al., 2012; Peyronnet et al., 2016; Ishikawa et al., 2018), the pathways responsible for the response, the mechanisms associated to its proarrhythmic effects, and their pharmacological modifications remain unclear (Neves et al., 2016).

Mechanical stretch increases Na^+^ influx into myocytes, involving (through the activation of the reverse mode of the Na^+^/Ca^2+^ exchanger) an increase in intracellular Ca^2+^ that lead to changes in cardiac electrophysiological properties favoring arrhythmia induction (Nattel and Dobrev, 2012; Neves et al., 2016). Several mechanisms have been implicated in the stretch-induced Na^+^ increase: stretch-activated channels (SACs) (K^+^ selective and cation non-selective channels) that modify Ca^2+^ and Na^+^ inflow, activation of the Na^+^/H^+^ exchanger, or mechanosensitivity of the voltage-gated sodium channels (Na_v_1.5) (Chorro et al., 2015; Peyronnet et al., 2016). Moreover, mechanical stretch stimulates the production of reactive oxygen species (ROS), which cause alterations in the Na_v_1.5 channels intensifying sodium inflow through the plateau of the action potential (Maltsev and Undrovinas, 2008; Prosser et al., 2013; Peyronnet et al., 2016). In fact, the late sodium inflow (*I*_NaL_) is produced by the delayed or incomplete inactivation of the Na_v_1.5 channel, and its augmentation has been described as a cause of after depolarizations and the triggering of arrhythmias (Song et al., 2008; Shryock et al., 2013; Belardinelli et al., 2015). In detail, the augmentation of late sodium current prolongs repolarization and facilitates the appearance of early after depolarizations, and the consequent Na^+^ overload is capable of causing delayed after depolarizations in atrial myocytes. Additionally, kinases such as Ca^2+^/calmodulin-dependent protein kinase (CaMKII), whose activity is enhanced by the stretch-induced increase in intracellular Ca^2+^, could modulate Na_v_1.5 channels (Ma et al., 2012; Shryock et al., 2013).

Ranolazine is an antianginal drug with confirmed beneficial effects against cardiac arrhythmias associated to the augmentation of *I*_NaL_ current and Ca^2+^ overload (Sossalla et al., 2010; Chorro et al., 2015; Caves et al., 2017; Karagueuzian et al., 2017). In fact, drugs capable of inhibiting *I*_NaL_, such as ranolazine, could reduce or suppress the occurrence of early and delayed after depolarizations and may be of therapeutic benefit to diminish the incidence of arrhythmias initiated by triggered activity (Song et al., 2008; Shryock et al., 2013; Karagueuzian et al., 2017). In this context, ranolazine has been described to reduce the incidence of non-sustained ventricular tachycardias and atrial fibrillation (AF) in patients with acute coronary syndrome without ST segment elevation (Gong et al., 2017). Its effects comprise (1) a decrease in the opening of the Na^+^ channels during the action potential upstroke (peak *I*_Na_) and plateau (late *I*_Na_), and (2) inhibition of the delayed rectifier potassium current (*I*_Kr_). In fact, these effects are directly related to the efficacy of ranolazine in the prevention of AF and in conversion to sinus rhythm (Gong et al., 2017; Patel and Kluger, 2018). Additionally, ranolazine can diminish or inhibit Na_v_1.5 mechanosensitivity, of relevance in alterations of mechano-electric dysfunction (Beyder et al., 2012; Strege et al., 2012). Therefore, the decrease in Na^+^ influx produced by ranolazine as a result of the action upon stretch-activated sodium currents or inhibition of the late *I*_Na_ activated by stretch-induced Ca^2+^ overload could attenuate or prevent the electrophysiological manifestations of mechanoelectric feedback.

The evaluation of the manifestations of mechanoelectric feedback and treatments that could attenuate or abolish them requires the development of experimental models that reproduce features of stretch-induced electrical modifications. Atrial murine immortalized cells (HL-1) exhibit spontaneous electromechanical activity whose characteristics have been shown to be sensitive to mechanical stimulation, and Na^+^ currents in these cells are responsive to ranolazine (Strege et al., 2012). In addition, it has been reported that confluent monolayers of HL-1 cells exhibit re-entrant conduction, making it possible to analyze re-entrant activation at the cellular level in order to study fibrillatory patterns under basal conditions and under pharmacological interventions (Hong et al., 2008; Climent et al., 2015; Houston et al., 2018; van Gorp et al., 2020).

The present study investigates the effects of mechanical stretch upon activation pattern characteristics in cultured atrial myocyte monolayers with the purpose of assessing the effect of ranolazine and determining whether its action could reduce the mechanoelectric feedback modifications.

## Materials and Methods

### Experimental Protocol

**FIGURE 1:**
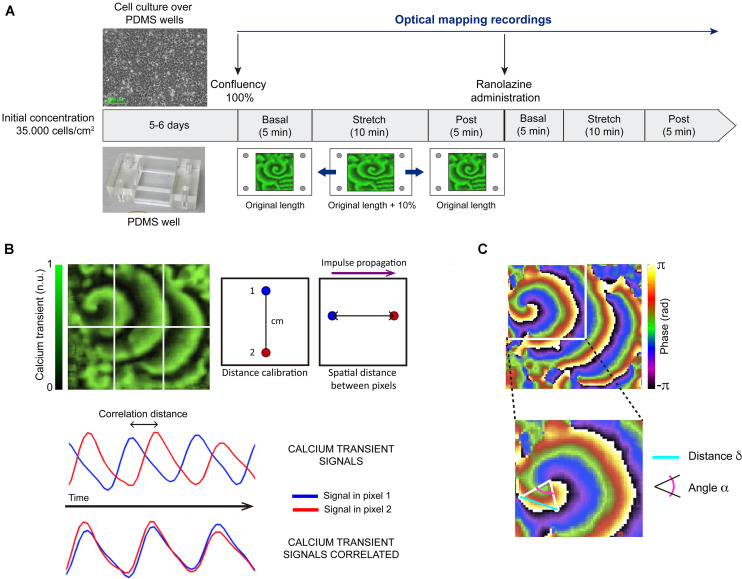
Summary of methodology. **(A)** Outline of the experimental protocol: after achieving cell confluence, optical mapping recordings were acquired each minute during the basal situation (5 min), under stretching to 110% of resting length (10 min), and after stretch suppression (5 min). The same protocol was repeated in the presence of ranolazine. **(B)** Process for calculating conduction velocity: calibration distance, pixel selection, correlation of fluorescence signal in time of pixel 1 and pixel 2, and conduction velocity obtained as the ratio spatial distance (cm)/correlation distance (s). n.u.: normalized units. **(C)** Approach for estimating curvature: transition lines from 0 to 2π selection, relative angle (α) and distance (δ) calculation, and curvature of the rotor measured as the spatial derivative of alpha (*d*α/*d*δ).

The experimental protocol was shown in Figure 1A. HL-1 cells were maintained, grown, and proliferated according to the protocol established by Claycomb et al. (1998) in 2 cm × 2 cm flexible polydimethylsiloxane wells (cell seeding density = 35,000 cell/cm^2^). After achieving further cell confluence (after 5–6 days of culture), HL-1 cells spontaneously presented fibrillatory activity (Climent et al., 2015; Del Canto et al., 2017). A total of 14 cell cultures were included in the study. In order to evaluate the effects of mechanical stretch on activation pattern characteristics, the cultured myocyte monolayers were stretched in a ST-CH-04 strain unit (Bridge), giving rise to 10% longitudinal increment along the horizontal axe (resting length: 2 cm, stretch length: 2.2 cm) for 10 min, and after this period, stretch was suppressed. The stretch percentage applied was according to previous studies where activation pattern changes were observed for a degree of mechanical stretch in the range of 10–15% (Tsai et al., 2011; Seo et al., 2014; Del Canto et al., 2018). The stretching was applied for 10 min in order to analyze the time evolution of stretch-induced effects, based on previous studies (Chorro et al., 2015; Del Canto et al., 2018).

Under control conditions (without drug), optical mapping recordings were acquired each minute during basal situation (5 min), under stretch (10 min), and after stretch suppression (5 min). The same protocol was repeated in the presence of ranolazine (Sigma-Aldrich, ref. R6152). A 1 mM stock solution of ranolazine in distilled water was prepared, ensuring the solubility criteria was met (H_2_O: <10 mg/ml). For the performance of the described protocol under ranolazine action, firstly, culture medium (Tyrode’s solution, whose composition is described in Calcium Dye Loading section) was changed to ranolazine solution (50 μM in Tyrode’s solution). After 5 min, the protocol was performed as previously described, maintaining superfusion during this phase of experiment protocol. The ranolazine concentration was within the range normally used in HL-1 cells studies (Strege et al., 2012). It should be noticed that, although the initial solution concentration was in human over therapeutic range, the estimated unbound concentration of the drug available to permeate cells is 18 μM, due to ranolazine protein binding (64%) (Jerling, 2006). Activity in the atrial myocyte monolayer was maintained throughout the experimental protocol, showing that cell viability was preserved under basal and stretch conditions.

In a second experimental series (*n* = 6), we carried out a time-matched control in order to analyze the behavior of the preparation in a second stretch. For this purpose, we performed the protocol described above in control conditions two times in a row: (1) basal (5 min), stretch (10 min), and post (5 min); (2) basal (5 min), stretch (10 min), and post (5 min).

### Calcium Dye Loading

For calcium transient (CaT) imaging, HL-1 cell cultures were stained by immersion in Claycomb cultures medium with rhod-2 AM (Ca^2+^-sensitive probe, TEFLabs, Inc., Austin, TX, United States) dissolved in DMSO (1 mM stock solution; 3.3 μl/ml in culture medium) and probenecid (TEFLabs, Inc., Austin, TX, United States) at 420 μM for 30 min under incubation conditions. Ca^2+^ transients are used as a surrogate for action potentials and to visualize propagation patterns due to the much larger fluorescence amplitude recorded compared to voltage dyes (Entcheva and Bien, 2006; Houston et al., 2018). This makes it possible to track wavefronts and to locate the reentry cores within monolayers. After dye incubation, culture medium was changed to fresh modified Tyrode solution at 36.5°C (containing, in mM: NaCl 120, NaHCO_3_ 25, H_2_O_4_PNa.H_2_O 1.2, MgCl_2_ 1, glucose 5.5, CaCl_2_ 1.8, KCl 5.4 and albumin 0.04 g/L). All chemicals were obtained from Sigma-Aldrich (Dorset, United Kingdom) or Fisher Scientific Inc. (New Jersey, United States).

### Optical Mapping

In order to excite rhod-2, cell cultures were illuminated with two filtered green LED light sources (CBT-90-G; peak output 58W; peak wavelength 524 nm; Luminus Devices, Billerica, MA, United States), with a plano-convex lens (LA1951; focal length = 25.4 mm; Thorlabs, Newton, NJ, United States) and a green excitation filter (D540/25X; Chroma Technology, Bellows Falls, VT, United States) (Climent et al., 2015). Fluorescence was recorded using an electron-multiplying charge-coupled device (EMCCD; Evolve-128: 128 × 128, 24 μm × 24 μm-square pixels, 16 bit; Photometrics, Tucson, AZ, United States) with a custom emission filter (ET585/50-800/200M; Chroma Technology) suitable for rhod-2 emission placed in front of a high-speed camera lens (DO-2595; Navitar Inc., Rochester, NY, United States). Fifteen-second movies of fluorescence were recorded at 100 frames/s throughout the protocol duration. The area of the field of view (FOV) was ∼2 cm × 2 cm (95 pixels × 95 pixels) and included the entire flexible well, both in control and stretch situations (Climent et al., 2015).

### Optical Data Processing and Analysis

Custom software written in MATLAB was used to perform optical mapping image processing and analysis (Climent et al., 2015). Prior to any analysis, raw data were masked in order to discard pixels outside the area of silicon wells and to always analyze a 2 cm × 2 cm area. Then, data were filtered to remove fluorescence noise, applying a spatial Gaussian filter (kernel size = 3) and a temporal smoothing filter (kernel size = 5). Finally, CaT signals of each pixel were normalized between 0 and 1 (Laughner et al., 2012).

#### Spectral Analysis

Power spectra of optical signals were estimated by using Welch periodogram (2-s Hamming window overlap). The dominant frequency (DF) of each pixel was determined as the frequency with the largest peak in the spectrum between 0.05 and 30 Hz (Berenfeld et al., 2000). For each individual cell culture, the highest DF was obtained as the maximum DF of the entire well. The regularity index (RI), defined as the ratio of the power within a 0.5-Hz band centered on the DF and the total power spectrum from 0.05 to 30 Hz, was determined for each pixel (Gutbrod et al., 2015). For each individual cell culture, the maximum RI was obtained from the pixels with the highest periodicity.

#### Phase Singularities and Rotor Dynamics

Phase maps of each movie were obtained by calculating the instantaneous phase of the Hilbert-transformed optical signals and phase singularities (PS) detection was performed according to methods described by Bray and Wikswo (2002). Once all PS were identified, they were connected in time and space into rotors; unstable rotors with durations of less than 100 ms were discarded. For each cell culture, we calculated the complexity of fibrillatory activity, defined as the mean number of simultaneous functional reentries (i.e., meandering reentries not around an anatomical obstacle) per square centimeter (i.e., PS/cm^2^). In addition, the meandering of each individual rotor was defined as the distance covered by the tip divided by the duration of the PS (i.e., cm/s) (Bray and Wikswo, 2002; Climent et al., 2015). Rotor meandering in each cell culture was calculated as the mean value of the meandering of all the PS detected during the recording, according to methodology described in Climent et al. (2015).

The estimation of rotor curvature was performed as described in previous publications (Climent et al., 2015). Firstly, the lines connecting phase transitions from 0 to 2π that originate at each rotor were selected. These transition lines were traced from the rotor tip to the periphery and the relative angle (α) and distance (δ) of line points with respect to the rotor tip were computed. Secondly, the curvature at each point in the transitional line was measured as the spatial derivative of α (*d*α/*d*δ). Finally, the curvature of the rotor was estimated as the mean value of curvature along the transitional line (Figure 1C).

#### Conduction Velocity

In order to obtain conduction velocity (CV) values, CaT maps were divided in 6 areas (up: left, center, right; down: left, center, right). In each area where the CaT impulse described a rectilinear path, two pixels were selected and their two optical signals over time were correlated. With a previously indicated calibration distance (spatial information) and the impulse correlation distance (temporal information), conduction velocity was calculated. Finally, the CV value of each map was computed as the average of determinations in each map area. This process can be observed in Figure 1B.

### Statistical Analysis

All values are presented as mean ± standard deviation (SD). A two-way analysis of variance (ANOVA) test or non-parametric Wilcoxon and Friedman’s tests were used to compare continuous variables, depending on the statistical distributions. Linear regression analysis was performed to evaluate the relationships between the complexity of fibrillatory activity and the DF, RI, conduction velocity, rotor curvature, and rotor meandering. The multivariate analysis was carried out using a stepwise multiple linear regression model. Differences were considered statistically significant when *p* < 0.05.

## Results

**FIGURE 2:**
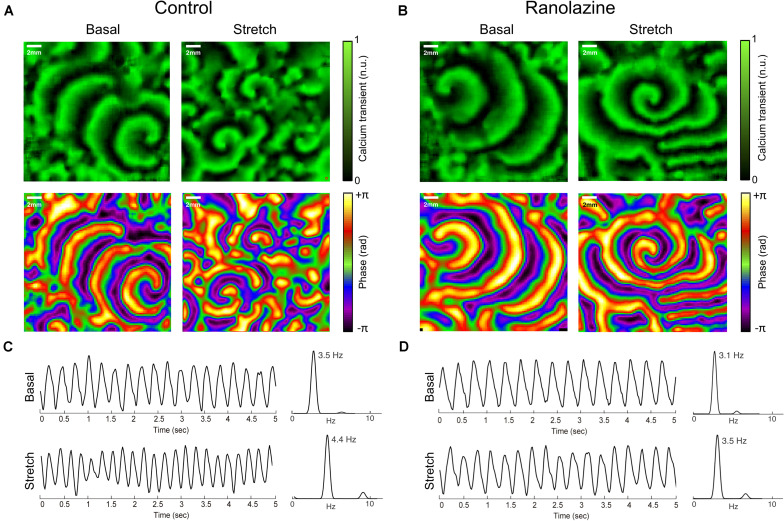
Overview of the effects of ranolazine on stretch-induced changes in atrial activation patterns. CaT (top) and phase (bottom) maps of representative cell cultures in basal situation (left) and during stretch (right), under control conditions **(A)**, and ranolazine action **(B)**. Time series of CaT optical signals and the corresponding power spectrum of the analyzed region in basal situation (top) and during stretch (bottom) for **(C)** control and **(D)** ranolazine conditions.

Figures 2A,B show normalized CaT and phase map images of representative cell cultures under control conditions (Figure 2A) and after the administration of ranolazine (Figure 2B), in basal and stretch situations. In this example, single rotors can be seen in CaT and phase map images in both basal (counterclockwise rotor) and stretch (clockwise rotor) situations, under ranolazine action (Figure 2B), whereas several wavebreaks and secondary rotors can be observed in stretched cells under control conditions (Figure 2A). Figures 2C,D show the time course of CaT and the corresponding power spectra in basal situation and during stretch for control (Figure 2C) and ranolazine (Figure 2D) conditions. It should be noticed that for this representative example, the activation rate of stretched cells was faster both under control conditions and after the administration of ranolazine—though acceleration was less pronounced under ranolazine action.

### Effects of Ranolazine on Baseline Fibrillatory Patterns

**FIGURE 3:**
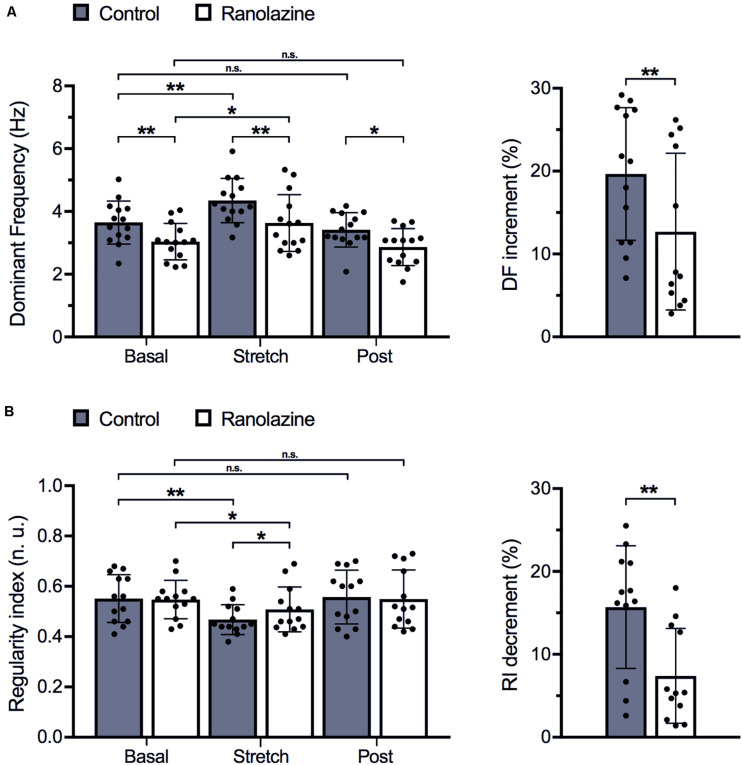
Effects of ranolazine on stretch-induced modifications of spectral characteristics of fibrillatory activity (mean ± standard deviation) (*n* = 14). **(A)** Dominant frequency under control and ranolazine conditions, for basal, stretch (3rd minute), and post-stretch (3rd minute) situations (left) and stretch-induced variation in dominant frequency under control conditions and under ranolazine action (right). **(B)** Regularity index under control and ranolazine conditions, for basal, stretch, and post-stretch situations (left) and stretch-induced variation in regularity index under control conditions and under ranolazine action (right). *p < 0.05, **p < 0.01, n.s. non-significance.

**FIGURE 4:**
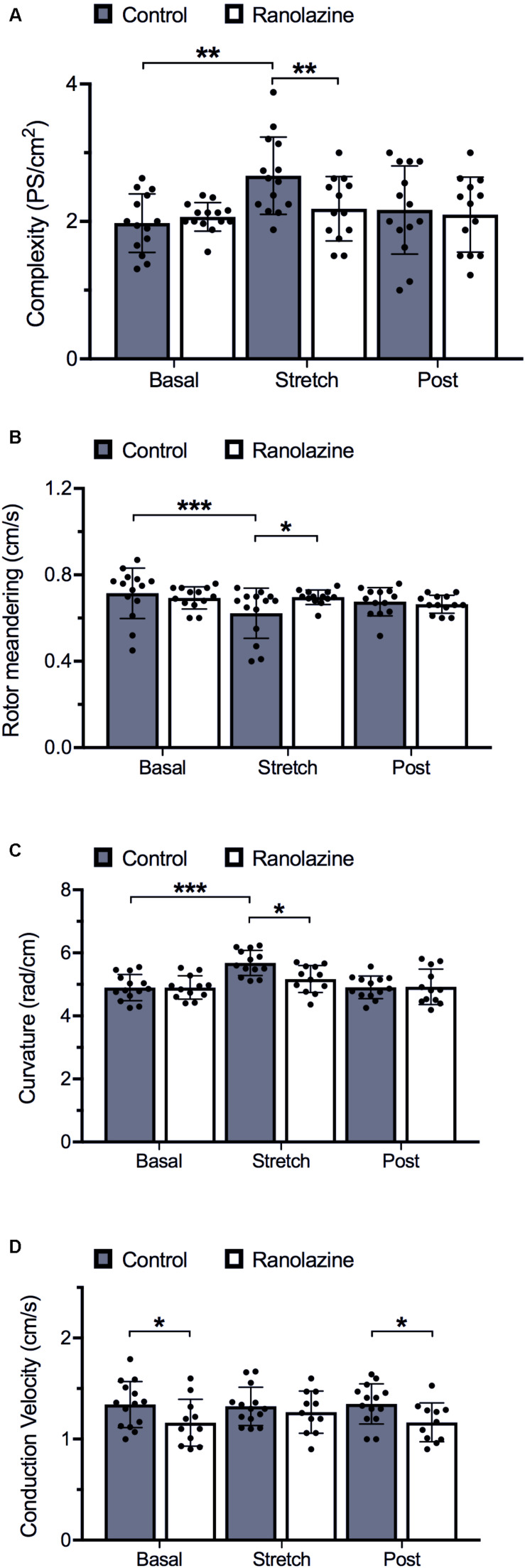
Quantification of activation pattern characteristics of cell cultures obtained under control and ranolazine conditions, for basal, stretch (3rd minute), and post-stretch (3rd minute) situations (mean ± standard deviation) (*n* = 14). **(A)** Complexity measured as the number of simultaneous phase singularities per square centimeter. **(B)** Rotor meandering (mean distance traveled for each rotor tip over time). **(C)** Rotor curvature. **(D)** Conduction velocity. *p < 0.05, **p < 0.01, ***p < 0.001.

In order to analyze the baseline (without stretch) patterns during fibrillatory activity and the modification induced by ranolazine, the characteristics of fibrillation were analyzed prior to and after suppressing stretch (Figures 3, 4)—all parameters being stable over time at baseline (variance < 0.25). Regarding the spectral characteristics, ranolazine reduced DF slowing the arrhythmia, i.e., increasing the fibrillatory cycle length, before stretch (3.04 ± 0.61 vs. 3.65 ± 0.69 Hz, *p* < 0.01) and after stretch suppression (2.87 ± 0.60 vs. 3.63 ± 0.31 Hz, *p* < 0.05) (Figure 3A). Nevertheless, RI showed no statistically significant variation under ranolazine effects (Figure 3B).

During baseline recordings, we observed that ranolazine did not significantly modify the complexity of propagation patterns of HL-1 cells; in fact, the number of singularity points per square centimeter was similar under ranolazine and control conditions (2.04 ± 0.21 vs. 1.97 ± 0.45 PS/cm^2^) (Figure 4A). Concerning rotor dynamics, rotor meandering (Figure 4B), and rotor curvature (Figure 4C) in non-stretched cell cultures likewise did not show statistically significant differences between both conditions. However, non-stretched cell cultures presented significantly lower CV under ranolazine action than under control conditions (pre-stretch: 1.17 ± 0.24 vs. 1.34 ± 0.22 cm/s, *p* < 0.05; post-stretch: 1.17 ± 0.19 vs. 1.35 ± 0.20 cm/s, *p* < 0.05) (Figure 4D).

### Effects of Ranolazine on Stretch-Induced Modifications of Activation Patterns

#### Spectral Characteristics

Figure 3 shows the effect of ranolazine on the stretch-induced modifications of spectral characteristics (DF—Figure 3A and RI—Figure 3B) for the entire series of experiments (*n* = 14). The performance of spectral analysis allowed localization of the moment of maximum effect of stretching (3 min after starting stretch) and the delay necessary for the disappearance of these effects (3 min after suppressing stretch), these three windows being the stages chosen to present the results of the study: basal (prior to stretch), during stretch (3rd minute), and post-stretch (3rd minute).

In the control series, stretch induced a significant increase in DF (3.65 ± 0.69 vs. 4.35 ± 0.74 Hz, *p* < 0.01) and a significant decrease in RI (0.55 ± 0.10 vs. 0.47 ± 0.06, *p* < 0.01). These parameters returned to baseline values after stretch suppression (DF: 3.63 ± 0.88 Hz, RI: 0.56 ± 0.11). Under ranolazine action, stretch-induced DF and RI modifications were attenuated (DF: 3.04 ± 0.61 vs. 3.42 ± 0.60 Hz, *p* < 0.05; RI: 0.55 ± 0.08 vs. 0.51 ± 0.10, *p* < 0.05). In fact, the magnitudes of the DF increment and RI decrement were smaller under ranolazine effects than under control conditions (DF: 12.8% vs. 19.7%, *p* < 0.01; RI: −7.4% vs. −15.3%, *p* < 0.01). Moreover, stretched cell cultures treated with ranolazine presented lower DFs (3.42 ± 0.18 vs. 4.35 ± 0.23 Hz, *p* < 0.01) and greater RI values (0.51 ± 0.10 vs. 0.47 ± 0.06, *p* < 0.05), consistent with the decreased activation rate and increased activation regularity induced by ranolazine.

**FIGURE 5:**
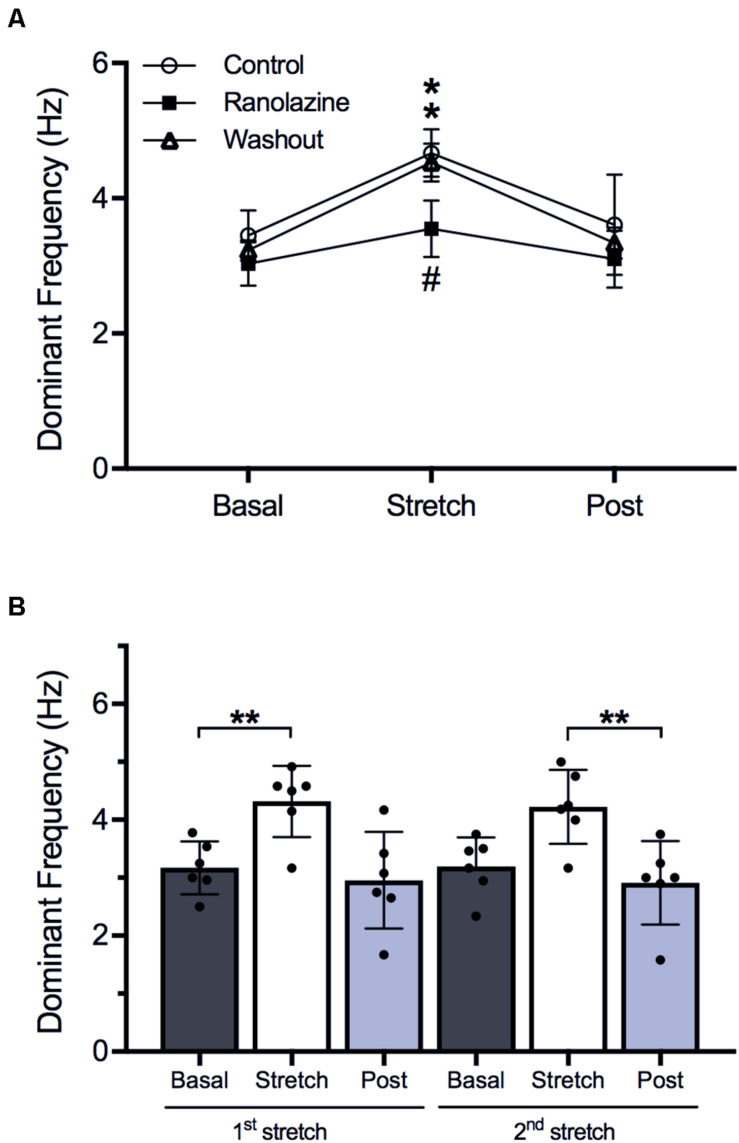
**(A)** Stretch-induced modifications of dominant frequency under control conditions, under ranolazine action, and after drug washout (*n* = 4). *p < 0.05 stretch vs. basal, ^#^*p* < 0.05 ranolazine vs. control and washout. **(B)** Effects of first and second stretch on dominant frequency (*n* = 6). **p < 0.01.

Additionally, in 4 of 14 cell monolayers, stretch protocol was repeated after washout of ranolazine in order to analyze the recovery of the drug effect. Figure 5A shows the stretch-induced changes in DF. Under control conditions, stretch induced a significant increase in DF (3.45 ± 0.37 vs. 4.67 ± 0.35 Hz, *p* < 0.05), and DF returned to baseline values after stretch suppression (3.61 ± 0.74 Hz). Under ranolazine action, the increase in DF was also significant (3.03 ± 0.32 vs. 3.55 ± 0.42 Hz, *p* < 0.05), but the magnitude of increment was smaller (control: 35%; ranolazine: 17%) and stretched cell cultures present lower DF value than in control conditions (3.55 ± 0.42 vs. 4.67 ± 0.35 Hz, *p* < 0.05). After drug washout, the stretch-induced changes in DF were similar to those obtained under control conditions (3.23 ± 0.15 vs. 4.53 ± 0.28 Hz, *p* < 0.05, increment: 39%).

On the other hand, the behavior of the preparation in a second stretch is shown in Figure 5B, where the values of DF through two consecutives stretches in the same preparation under control conditions are presented. During the first stretch, DF increased (3.17 ± 0.46 vs. 4.32 ± 0.61 Hz, *p* < 0.01) and returned to baseline values after suppressing it (2.96 ± 0.84 Hz). During the second stretch, DF increased (3.19 ± 0.50 vs. 4.23 ± 0.64 Hz, *p* < 0.01) and returned to baseline values (2.91 ± 0.72 Hz). No significant differences were observed between the modifications produced by the first and the second stretch.

#### Rotor Dynamics and Fibrillation Complexity

Figure 4A shows the effect of stretch on activation complexity under control conditions and after the administration of ranolazine. In the control series, stretch significantly modified the complexity of the CaT propagation patterns of HL-1 cells during the arrhythmia. The number of singularity points per square centimeter was significantly higher in the stretch than in the basal situation (1.97 ± 0.45 vs. 2.66 ± 0.58 PS/cm^2^, *p* < 0.01). Figure 2A shows CaT and phase map images of representative cell cultures under control conditions in basal and stretch situations. In the baseline example (left), a single rotor located in the lower-right region of the membrane plate generated relatively regular wavefronts that covered most of the region, whereas in the stretched cells phase map (right), several small wavefronts and PS were observed (Supplementary Video S1). However, under ranolazine action, complexity did not increase during stretch (Figure 4A). In addition, for the stretch situation, fibrillation complexity after ranolazine infusion was significantly lower than under control conditions (2.18 ± 0.49 vs. 2.66 ± 0.58 PS/cm^2^, *p* < 0.05). In fact, as shown in the phase maps snapshots (Figure 2B), the number of simultaneous rotors was significantly reduced following ranolazine administration (Supplementary Video S2).

**FIGURE 6:**
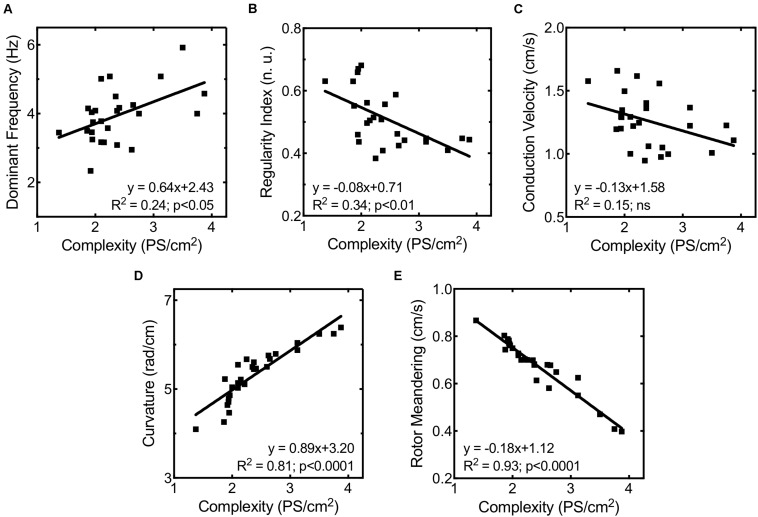
Correlation between fibrillation complexity and **(A)** highest dominant frequency, **(B)** maximum regularity index, **(C)** mean conduction velocity, **(D)** rotor curvature, and **(E)** rotor meandering.

In order to evaluate the mechanisms responsible for increased fibrillation complexity in stretched cell cultures, mean CV, rotor curvature, and rotor meandering were measured and compared between situations (basal vs. stretch). Figure 6 shows the regression lines obtained on relating the activation complexity to DF, RI, CV, rotor curvature, and rotor meandering. We found DF, RI, and CV to exhibit a weak correlation to complexity (*R*^2^ = 0.24, *R*^2^ = 0.34, and *R*^2^ = 0.15, respectively, Figures 6A–C), whereas parameters associated with rotor dynamics (wavefront curvature and rotor meandering) showed a significant correlation to activation complexity (*R*^2^ = 0.89 and *R*^2^ = 0.81, respectively; Figures 6D,E). As observed in Figures 4B,C, rotor dynamics were modified by stretch under control conditions: average meandering decreased significantly (0.72 ± 0.12 vs. 0.62 ± 0.12 cm/s, *p* < 0.001) (Figure 4B), and this change in rotor tip movement was associated with an increase in the wavefront curvature (4.90 ± 0.42 vs. 5.68 ± 0.40 rad/cm, *p* < 0.001) (Figure 4C). These modifications in rotor dynamics decreased the area needed for each rotor to be self-sustained and could explain the stretch-induced increment in complexity (Figure 4A). In contrast, no significant differences in CV were observed in the stretch situation with respect to baseline (Figure 4D).

The administration of ranolazine attenuated the normal stretch-induced effects, resulting in lesser fibrillation complexity in stretched cell cultures (Figure 4A). This reduction in the complexity was not related to significant modifications of CV; in fact, no significant differences in CV were found in stretched cells between the control and ranolazine conditions (Figure 4D). However, ranolazine increased rotor meandering in stretched cell cultures (0.70 ± 0.03 cm/s, *p* < 0.05 vs. control) (Figure 4B) and reduced wavefront curvature (5.17 ± 0.43 rad/cm, *p* < 0.05 vs. control) (Figure 4C)—these effects being associated with lesser fibrillation complexity (Figures 6D,E).

Furthermore, the multiple linear regression analysis using the activation complexity as dependent variable only introduced rotor curvature as an independent variable in the function (*R*^2^ = 0.85; *p* < 0.0001; standard error of estimate = 0.22).

## Discussion

In this study, we used an experimental model involving HL-1 cell monolayers to analyze the characteristics of activation patterns during fibrillatory activity in order to describe the stretch-induced modifications and analyze the effects of ranolazine upon the responses induced by mechanical stretch.

### Main Findings

**FIGURE 7:**
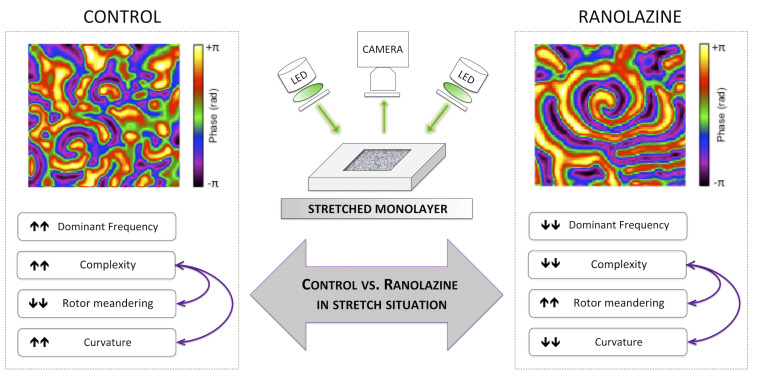
Summary of the main findings of the study: effects of the inhibition of late sodium current by ranolazine on activation patterns in stretched atrial cell monolayers.

The main findings of this study are that ranolazine attenuates the increase in activation frequency and complexity of the arrhythmia induced by mechanical stretch in HL-1 cells. In addition, our results suggest that this protective effect is associated with a modification of rotor dynamics in stretched cell cultures under ranolazine action (Figure 7).

### Effects of Ranolazine on Baseline Fibrillation Characteristics

Myocardial activation during fibrillatory activity is complex (Salinet et al., 2017; Meo et al., 2018). In the present study, in the determinations made in the absence of stretch, we observed a slowing effect on activation frequency during the arrhythmia by ranolazine. This effect could be associated to the increase in refractoriness produced by the drug, due to repolarizing I_Kr_ block effect and a prolongation of the recovery of Na^+^ channels (post-repolarization refractoriness) (Quinn and Kohl, 2016; Caves et al., 2017; Karagueuzian et al., 2017). In previous studies, DF has shown an inverse correlation to the electrophysiological parameters related to ventricular refractoriness (Chorro et al., 2015; Del Canto et al., 2018). Moreover, the lower activation frequency observed is related to a reduction of CV as a consequence of the inhibition of peak *I*_Na_ (Berenfeld et al., 2000; Caves et al., 2017). Previous experimental studies have reported a peak *I*_Na_ blocking effect of ranolazine in atrial cells, secondary to a significantly negative shift in voltage of half-maximal inactivation and slower recovery from inactivation of *I*_Na_ (Beyder et al., 2012; Caves et al., 2017).

However, this slowing effect was not accompanied by a reduction in the complexity of the arrhythmia that could be explained by the reduction in the CV observed under ranolazine action in no-stretch cells. Nevertheless, we did not observe a greater complexity most likely because in this situation (no-stretch) both effects of ranolazine (decrease in CV and increase in refractoriness) are canceled out.

### Modification of the Stretch-Induced Effects by Ranolazine

In the present work, stretch increased DF and activation complexity of Ca^2+^ transients propagation patterns. It also produced a decrease in the RI of the recordings, indicating more disorganized activation during the arrhythmia.

The increase in fibrillation complexity in stretched cell cultures under control conditions was associated to a decrease in rotor meandering and an increase in reentrant wavefront curvature, allowing the formation of more simultaneous rotors. Additionally, it has been reported that the increase in DF is related to a reduction in the time required by a rotor to complete a period, mainly due to reduction of the area of reentry (i.e., rotor meandering), observed in action potential propagation (Pandit and Jalife, 2013) and in CaT propagation patterns (Climent et al., 2015). In fact, a reduction in tip meandering allows faster circumscription of the core of the rotor. These stretch effects are frequently associated to shorter refractoriness (Chorro et al., 2015; Del Canto et al., 2018), which is consistent with previous experimental studies in isolated rabbit hearts that showed a stretch-induced decrease in refractory periods and action potential duration (Ravelli and Allessie, 1997; Quinn and Kohl, 2016; Del Canto et al., 2018). Shortening of action potential duration has been related to stretch-induced increased intracellular Na^+^ accumulation through several mechanisms: (1) enhancement of outward current through the Na^+^/K^+^ pump and the reverse mode of the Na^+^/Ca^2+^ exchanger; and (2) modulation of outward K^+^ currents (increase) and *I*_CaL_ (inhibition) in response to the increase in intracellular Ca^2+^ concentration, following activation of the reverse mode of the Na^+^/Ca^2+^ exchanger (Nattel and Dobrev, 2012). Furthermore, the modulation of some outward K^+^ currents (*I*_K__1_) has been associated with a reduction in rotor meandering and a decrease in the area required to maintain the reentry, effects reported in action potential propagation patterns (Pandit et al., 2005) and in CaT activation patterns (Climent et al., 2015). Another possible mechanism for explaining higher DF and complexity is the increase in the electrophysiological heterogeneity produced by stretch (Pandit and Jalife, 2013), as evaluated by the RI of the arrhythmia.

The administration of ranolazine reduced the stretch-induced changes on activation patterns upon HL-1 cells. In fact, DF increments and RI decrements under stretch were smaller under ranolazine action. Moreover, complexity did not increase in stretched cell cultures under ranolazine action and was lower than under control conditions. Such lower DF and complexity of arrhythmia may be explained by the modification in rotor dynamics induced by ranolazine in stretched cell cultures: (1) the increasing of area reentry (rotor meandering) implies a longer rotation period in the absence of CV modifications and consequently a reduction in DF; (2) this greater rotor tip meandering, together with a decrease in the rotor curvature, involves widening of the area required by a rotor to be self-sustained, and thus the number of rotors per area unit was reduced (i.e., complexity). Additionally, the lower heterogeneity (i.e., RI) observed in stretched cell cultures treated with ranolazine may contribute to the reduction in complexity and activation rate.

Attenuation of the stretch-induced effects under ranolazine could be explained by different mechanisms. It has been described that stretch elevates intracellular Na^+^ and Ca^2+^, and this increase alters the redox state and modulates several ionic currents related to shortening of action potential duration and refractoriness: an increase in outward current through the Na^+^/K^+^ pump, an enhancement of Ca^2+^-dependent K^+^ currents, and a decrease in *I*_Ca__L_ (Nattel and Dobrev, 2012). Moreover, the Ca^2+^ overload induces Ca^2+^ release from the sarcoplasmic reticulum, leading to Ca^2+^ processes involved in AF-related mechanisms (focal ectopic activity, as delayed after depolarizations) (Nattel and Dobrev, 2012; Shryock et al., 2013; Belardinelli et al., 2015). Additionally, these stretch-induced modifications (increased intracellular Ca^2+^ and production of ROS) augment the persistent sodium inflow through the plateau of the action potential (*I*_NaL_) (Maltsev and Undrovinas, 2008; Ma et al., 2012; Karagueuzian et al., 2017), which contributes to increase intracellular Na^+^. Ranolazine inhibits *I*_Na__L_ (Antzelevitch et al., 2011), and this effect may normalize stretch-induced Na^+^ entry to myocytes and the resulting increase in cytosolic Ca^2+^ (through reverse mode Na^+^/Ca^2+^) that also enhances *I*_NaL_ (Ma et al., 2012; Karagueuzian et al., 2017). Accordingly, the regulation of increases in sarcoplasmic Ca^2+^ could prevent Ca^2+^-induced alterations, such as shortening of refractoriness and focal ectopic activity, involved in the mechanisms underlying AF (Song et al., 2008; Nattel and Dobrev, 2012; Shryock et al., 2013). In previous investigations in isolated hearts, comparable effects on stretch-induced activation patterns remodeling have been described (Chorro et al., 2015; Del Canto et al., 2018). These previous observations, together with the results obtained in our study, seem to confirm the role of late sodium current in the mechanisms of stretch.

However, these conclusions should take into account that the observations of the present work are based on the activation patterns of CaT, which, although they are consistent with the action potential propagation patterns (Houston et al., 2018), do not allow concluding directly on the electrophysiological effects of mechanical stretch.

On the other hand, as previously mentioned, the inhibition of the late sodium current by ranolazine implies a reduction of calcium influx (through reverse mode Na^+^/Ca^2+^) and this modulation would prevent the increase of Ca^2+^-dependent potassium currents, associated with a reduction in the rotor meandering and a decrease in the area required to maintain reentry. Therefore, the effects of ranolazine on Na^+^ channels would avoid the modification of the rotors that underlies the increase in complexity induced by stretching.

Another factor that may be related to the effects of ranolazine in HL-1 cells during stretch is the action of the drug upon the stretch-induced modulation of Na_v_1.5. The mechanosensitivity of Na_v_1.5 consists of accelerated kinetics, an increased peak current and stabilization of inactivation. However, modulation of mechanosensitivity of Na_v_1.5 has been demonstrated at higher ranolazine concentrations, though the drug effects depend on the magnitude of stretch and on the ranolazine membrane concentration (Beyder et al., 2012; Strege et al., 2012).

On the other hand, as reported in the literature, ranolazine at high concentration (>40 μM) is able to block the peak sodium current in atrial cells (Nesterenko et al., 2011; Caves et al., 2017). In the present study, our results demonstrated that ranolazine did not modify CV in stretched atrial monolayers. Therefore, we could consider that the peak Na^+^ current blocking action of ranolazine does not seem to be directly involved in the attenuation of stretch-induced electrophysiological effects.

Furthermore, ranolazine inhibits other ionic currents such as *I*_CaL_ and *I*_Na–Ca_, which are some of the mechanisms involved in the effects of stretch (Chorro et al., 2015); however, the inhibition of these currents was weak in atrial cells at the concentration used in the present study (IC_50_ ∼300 and ∼100 μM, respectively) (Antzelevitch et al., 2011).

Finally, other mechanisms that can be involved in the stretch effects are SACs, which serve as cardiac mechano-transducers increasing the influx of Na^+^ and Ca^2+^ to cardiomyocytes (Quinn and Kohl, 2016). However, in HL-1 cells, it has been reported that it is less likely that non-selective cation stretch-sensitive channels (SACs) are involved in the stretch response (Strege et al., 2012), suggesting that other possible mechanisms could be responsible for the stretch-induced effects.

In summary, the results of the present study support the hypothesis that the *I*_NaL_ blocker ranolazine could modulate cardiomyocyte mechanosensitivity and reduce the arrhythmogenic effects of stretch, suggesting a possible protective role of the inhibition of *I*_NaL_ in this context. However, further studies analyzing more preferential blockers are needed to clarify the role of late sodium current in mechanisms involved in mechanoelectric feedback.

### Study Limitations

The present study was performed using a specific experimental model of stretch based on HL-1 cells, which present a differentiated genotype and phenotype with characteristics of adult atrial myocytes (Claycomb et al., 1998; White et al., 2004; Dias et al., 2014). HL-1 cells constitute a well-characterized atrial myocyte culture line, widely used to investigate cardiac electrophysiology and arrhythmias (Climent et al., 2015; van Gorp et al., 2020). Nevertheless, extrapolation of the results obtained in the present study should consider the differences between HL-1 cell cultures and human atrial tissue, and the characteristics of the experimental preparation for inducing acute stretch, since the effects of stretch in chronic preparations (e.g., 24 h) and *in situ* atrium preparations can lead to different responses (Chorro et al., 2015; Del Canto et al., 2018).

On the other hand, as mentioned above, the characteristics of this experimental model do not allow the study of activation patterns during programmed electrical stimulation, because the area of cultured monolayer facilitated the spontaneous fibrillatory activity (van Gorp et al., 2020; Climent et al., 2015; Agladze et al., 2017). However, it should be noted that, despite these disadvantages, HL-1 cultures have been previously used to study the effect of certain manipulations and drugs on the complexity of fibrillatory conduction (Climent et al., 2015; van Gorp et al., 2020).

Another important limitation is the use of CaT as a surrogate for action potentials, an approach that requires caution in obtaining conclusions from the observations made and their relation to changes in electrophysiological properties. Nevertheless, the study of activation patterns and its rotor dynamics is possible by tracking wavefronts and locating the reentry cores within monolayers (Houston et al., 2018).

Besides, the complexity of activation in HL-1 monolayers and the presence of multiple reentrant waves can give rise to inaccuracies in conduction velocity determination. Therefore, to avoid or mitigate these limitations, CV was calculated considering those areas in activation maps where the electrical impulse described a rectilinear path.

Finally, in the present investigation, the effect of ranolazine on the electrophysiology and rotor dynamics of stretched atrial substrate have been investigated in a 2D monolayer model, which represents a simplified model of the 3D characteristics found *in vivo*. However, it has been reported that impulse propagation through gap junctions occurs in this cell model in the same manner as in more complex models, displaying reentrant wavefronts (rotor dynamics) and allowing the study of fibrillatory patterns under basal conditions or under pharmacological interventions (Hong et al., 2008; Climent et al., 2015; Houston et al., 2018).

## Conclusion

Ranolazine attenuates the electrophysiological effects responsible for the stretch-induced alterations in HL-1 cell fibrillatory activation patterns. In addition, our results suggest that modifications in rotor dynamics underlie the increased complexity of stretched cell cultures, and therefore, the protective effect of ranolazine would be associated with its counteraction upon these modifications. These observations may help to explain the mechanisms involved in mechanoelectric feedback, attributing a possible protective role to the modulation of Na^+^ channel by ranolazine.

## Data Availability Statement

The raw data supporting the conclusions of this article will be made available by the authors, without undue reservation, to any qualified researcher.

## Author Contributions

ID-C, FA, FF-A, FC, and AC conceived and designed the research. ID-C, LG-C, and IH-R performed the experiments. ID-C, LG-C, and MG analyzed the data. ID-C, LG-C, MG, FA, FF-A, FC, and AC interpreted results of experiments. ID-C prepared figures. ID-C and AC drafted the manuscript. ID-C, LG-C, IH-R, MG, MF-S, FA, LS, FF-A, FC, and AC edited and revised the manuscript and approved the final version of the manuscript. All authors contributed to the article and approved the submitted version.

## Conflict of Interest

The authors declare that the research was conducted in the absence of any commercial or financial relationships that could be construed as a potential conflict of interest.

## Supplementary Material

VIDEO S1 (file “MapIm_1.mov”) CaT and phase map images of representative cell cultures under control conditions in basal and stretch situations.

VIDEO S2 (file “MapIm_2.mov”) CaT and phase map images of representative cell cultures under ranolazine action in basal and stretch situations.
